# ARNI in HFrEF—One-Centre Experience in the Era before the 2021 ESC HF Recommendations

**DOI:** 10.3390/ijerph19042089

**Published:** 2022-02-13

**Authors:** Rafał Niemiec, Irmina Morawska, Maria Stec, Wiktoria Kuczmik, Andrzej S. Swinarew, Arkadiusz Stanula, Katarzyna Mizia-Stec

**Affiliations:** 1 Upper Silesian Medical Centre, First Department of Cardiology, School of Medicine in Katowice, Medical University of Silesia, 40-055 Katowice, Poland; irmina.morawska@gmail.com; 2 Upper Silesian Medical Centre, Students’ Scientific Society of the First Department of Cardiology, School of Medicine in Katowice, Medical University of Silesia, 40-055 Katowice, Poland; mariaannastec@gmail.com (M.S.); wikikuczmik@interia.pl (W.K.); 3 Faculty of Computer Science and Material Science, Institute of Material Science, University of Silesia in Katowice, 40-055 Katowice, Poland; andrzej.swinarew@us.edu.pl; 4 Institute of Sport Sciences, The Jerzy Kukuczka Academy of Physical Education, 40-065 Katowice, Poland; a.stanula@awf.katowice.pl

**Keywords:** heart failure, heart failure with reduced left ventricular ejection fraction, sacubitril/valsartan, HF, HFrEF, ARNI

## Abstract

Background: Sacubitril/valsartan, an angiotensin receptor–neprilysin inhibitor (ARNI), has demonstrated a survival benefit and reduces heart failure hospitalization in patients with heart failure with reduced left ventricular ejection fraction (HFrEF); however, our experience in this field is limited. This study aimed to summarize a real clinical practice of the use of ARNI in HFrEF patients hospitalized due to HFrEF in the era before the 2021 ESC HF recommendations, as well as assess their clinical outcome with regard to ARNI administration. Methods and Materials: Overall, 613 patients with HFrEF hospitalized in 2018–2020 were enrolled into a retrospective one-centre cross-sectional analysis. The study population was categorized into patients receiving (82/13.4%) and not-receiving (531/82.6%) ARNI. Clinical outcomes defined as rehospitalization, number of rehospitalizations, time to the first rehospitalization and death from any cause were analysed in the 1–2 year follow-up in the ARNI and non-ARNI groups, matched as to age and LVEF. Results: Clinical characteristics revealed the following differences between ARNI and non-ARNI groups: A higher percentage of cardiovascular implantable electronic devices (CIED) (*p* = 0.014) and defibrillators with cardiac resynchronization therapy (CRT-D) (*p* = 0.038), higher frequency of atrial fibrillation (*p* = 0.002) and history of stroke (*p* = 0.024) were in the ARNI group. The percentage of patients with HFrEF NYHA III/IV presented an increasing trend to be higher in the ARNI (64.1%) as compared to the non-ARNI group (51.5%, *p* = 0.154). Incidence of rehospitalization, number of rehospitalizations and time to the first rehospitalization were comparable between the groups. There were no differences between the numbers of deaths of any cause in the ARNI (28%) and non-ARNI (28%) groups. The independent negative predictor of death in the whole population of ARNI and non-ARNI groups was the coexistence of coronary artery disease (CAD) (beta= −0.924, HR 0.806, *p* = 0.011). Conclusions: Our current positive experience in ARNI therapy is limited to extremely severe patients with HFrEF. Regardless of the more advanced HF and HF comorbidities, the patients treated with ARNI presented similar mortality and rehospitalizations as the patients treated by standard therapy.

## 1. Introduction

Pharmacotherapy in heart failure with reduced ejection fraction (HFrEF) has evolved over the past decades. In 2014, a new molecule, LCZ696, was presented in the global PARADIGM-HF randomized trial, which is a combination of the neprilysin inhibitor (sacubitril) with the angiotensin II receptor blocker (ARB) (valsartan) [1,2]. It created a new group of drugs named angiotensin receptor–neprilysin inhibitor (ARNI); however, it needed 8 years before ARNI became the first line in HFrEF [3]. During this period, the levels of recommendations for the use of ARNI had been increasing. ESC 2016 Guidelines for the diagnosis and treatment of acute and chronic heart failure included sacubitril/valsartan in the therapeutic algorithm, which is recommended as a replacement for ACEI in ambulatory HFrEF patients who are symptomatic despite optimal therapy and who fit the PARADIGM-HF trial criteria [3].

In the following years, new expert recommendations were published suggesting the use of ARNI in a wider group of patients. In 2019, the ESC expert consensus considered initiation of ARNI rather than an ACE inhibitor or ARB for patients hospitalized with new-onset HF or acute decompensated chronic HF (ADHF) [4]. In 2021, the consensus of ACC (American College of Cardiology) experts was that ARNI takes the first position in the treatment algorithm of patients with HFrEF as the preferred option as an antagonist of the renin–angiotensin–aldosterone system (RAAS) prior to the use of ACEI or ARB [5]. Currently, it is also recommended to consider initiation of sacubitril/valsartan in ACEi naïve (de novo) patients with HFrEF [3].

Sacubitril/valsartan has been available in Poland since 2017. Unfortunately, despite the many benefits and significant advantages of ARNI over standard care, only a small part of the eligible patients has received the drug. ARNI has been administered based on the latest medical reports, recommendations and the results of a randomized controlled trial (RCT).

We were aware of the advantages of ARNI administration: the clinical improvement observed in consecutive patients as well as their positive opinion.

In this article, we want to summarize a real clinical practice of the use of sacubitril/valsartan in HFrEF patients hospitalized before the publication of the 2021 ESC Guidelines, as well as assess the clinical outcome of HFrEF patients with regard to ARNI administration.

## 2. Materials and Methods

### 2.1. Study Population

The study was conducted as a retrospective one-centre cross-sectional analysis of 1065 consecutive hospitalizations for HF. Into analysis, we included patients with a diagnosis of HF using the *International Classification of Diseases* (*ICD*) billing codes (10th edition, I50.X). The analysis comprised patients hospitalized in the I Department of Cardiology, the Medical University of Silesia in Katowice from January 2018 to December 2020. The data were obtained from electronic medical records.

In further analysis, we included only the hospitalization due to HFrEF. In this way, we obtained a group of 613 patients hospitalized due to HF symptoms, either for the first time for HF diagnosis and treatment initiation or optimization of the previously diagnosed HFrEF.

Taking into regard ARNI administration, we differentiated two subpopulations: 82 patients receiving sacubitril/valsartan (ARNI subpopulation) and 531 patients not receiving sacubitril/valsartan (non-ARNI subpopulation), treated with renin–angiotensin–aldosterone system (RAAS) inhibitors.

ARNI subpopulation included 28 patients already undergoing sacubitril/valsartan therapy and 54 patients for whom sacubitril/valsartan was prescribed for the first time during the current hospitalization.

All patients were compensated at discharge and received an optimal medical therapy of HFrEF, including neurohormonal antagonists (ACEIs/ARB or ARNI, MRAs and beta-blockers) and diuretics in patients with symptoms and/or signs of congestion [3]. Considering clinical status and recommendations, an invasive coronary angiography and optimal revascularization were performed in patients with coronary artery disease (CAD).

### 2.2. Matching

Taking into regard differences in the age and left ventricle ejection fraction (LVEF) between the whole ARNI and non-ARNI subpopulations, the matching analysis was carried out.

Finally, two groups of patients with HFrEF (ARNI group and non-ARNI group) were obtained, each consisting of 64 patients. These two groups were further analysed to compare the baseline characteristics and clinical outcomes. Clinical outcomes were defined as rehospitalization, number of rehospitalizations, time to the first rehospitalization and death from any cause. Median follow up was 17.5 (8–27.5) months.

### 2.3. Statistical Analysis

The study population was first dichotomized into 2 groups, receiving and non-receiving sacubitril/valsartan. Clinical characteristics and outcomes were compared between groups. Continuous variables were presented as mean ± standard deviation or median (1–3 quartile) and categorical as absolute values and percentages. Normality was verified using the Shapiro–Wilk test. The comparisons of groups were based on student’s two-sample t-tests or nonparametric Mann–Whitney U tests, as appropriate. The differences in proportions between groups were analysed using the χ^2^ test. A *p*-value ≤ 0.05 was considered statistically significant for all tests. Matching was performed by using the 1:1 nearest neighbour (NN) method without returning using R statistical environment extended via the MatchIt package. All other analyses were performed using MedCalc^®^ version 20.015 software.

## 3. Results

### 3.1. Study Population and Matching Results

**Figure 1:**
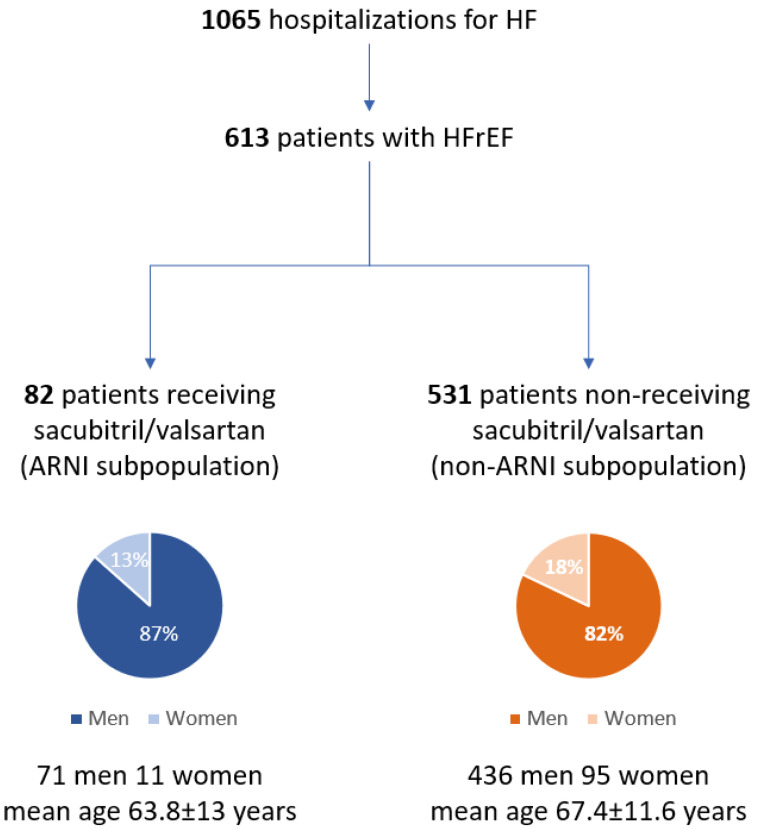
Flowchart of the whole population of patients hospitalized due to heart failure (I Department of Cardiology, Medical University of Silesia in Katowice, January 2018 to December 2020). HF: heart failure; HFrEF: heart failure with reduced ejection function; ARNI: angiotensin receptor–neprilysin inhibitor.

The study population consisted of 613 patients with HFrEF (507M, mean age 67.1 ± 11.7 years) among whom two subpopulations were distinguished: ARNI subpopulation (13.4%, 71 men, mean age 63.8 ± 13 years) and non-ARNI subpopulation (82.6%, 436 men, mean age 67.7 ± 11.4 years) (Figure 1).

The compared values of the study population before and after matching are detailed in Table 1.

### 3.2. Sacubitril/Valsartan Administration in Patients with HFrEF

**Figure 2:**
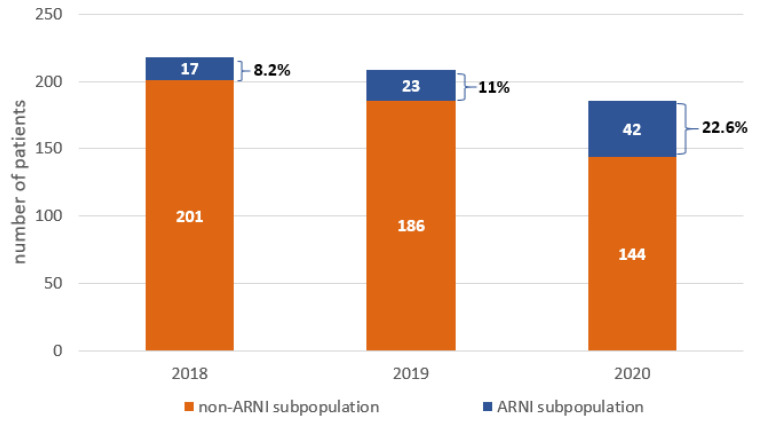
The number of hospitalizations and sacubitril/valsartan usage in patients with heart failure with reduced ejection function (HFrEF) in 2018–2020. ARNI: angiotensin receptor–neprilysin inhibitor.

Among the 613 patients hospitalized due to HFrEF, only 82 (13.4%) patients were treated using ARNI. The percentage of these patients was increased from 2018–2020. In 2018, only 17 (7.8%) received ARNI, in 2019 only 23 (11%) and in 2020 only 42 (22.6%) patients were treated with ARNI (Figure 2).

### 3.3. Comparison of Baseline Characteristic of ARNI and Non-ARNI Group

The following differences in clinical characteristics between ARNI and non-ARNI groups were observed: A higher percentage of the implanted CIED (*p* = 0.014) and CRT-D (*p* = 0.038), higher frequency of atrial fibrillation (AF) (*p* = 0.002) and history of stroke (*p* = 0.024) in ARNI group. The percentage of patients with HFrEF NYHA III/IV presented an increasing trend to be higher in ARNI (64.1%) as compared to the non-ARNI group (51.5%, *p* = 0.154) (Table 2).

### 3.4. Clinical Out-Comes in ARNI and Non-ARNI Groups

#### 3.4.1. Rehospitalization

Both incidences of rehospitalization and the number of rehospitalizations were similar for both ARNI and non-ARNI groups: 12 patients in the ARNI subgroup and 13 patients in the non-ARNI group required rehospitalization. The time to the first rehospitalization was also comparable between the groups: non-ARNI: 126 (83.8; 209.8) days vs. ARNI: 114.5 (71; 268.5) days (Table 1).

#### 3.4.2. Total Mortality

There were no differences between the numbers of deaths of any cause in the ARNI (18/28%; 17 males, 1 female) and non-ARNI (18/28%, 18 males) groups.

The clinical characteristics of the patients who died during follow-up revealed a higher prevalence of coronary artery disease (CAD) in ARNI patients (83.3 vs. 33.3%, *p* = 0.008) (Table 1.).

The independent negative predictor of death in the whole population of ARNI and non-ARNI groups was the coexistence of CAD (beta= −0.924, HR 0.806, *p* = 0.011).

#### 3.4.3. ARNI and Non-ARNI Groups: Clinical Characteristic of Death vs. Alive Patients

ARNI patients who died during observation were older (66.6 ± 13.9 vs. 62.5 ± 11.4 years, *p* = 0.007), had lower LVEF (20.2 ± 7.1 vs. 25.1 ± 10.3%, *p* = 0.048), presented more advanced NYHA class (*p* = 0.049) and lower frequency of atrial fibrillation. There was a non-significant difference between the frequency of CAD and chronic obstructive pulmonary disease (COPD): higher among dead patients (Table 3).

Non-ARNI patients who died during observation presented lower LVEF (20.4 ± 7.5 vs. 24.8 ± 8.4% *p* = 0.050), more advanced NYHA class (*p* = 0.030), higher frequency of tricuspid regurgitation II/III (50% vs. 23.0%, *p* = 0.045) and lower frequency of systemic hypertension (27.8% vs. 67.4%, *p* = 0.004) (Table 4).

Clinical data analysis of the ARNI vs. non-ARNI patients who died revealed a higher frequency of CAD in ARNI subjects (83.3 vs. 33.3%, *p* = 0.003) only. There was a trend towards longer time to the first rehospitalization in the ARNI as compared to the non-ARNI subjects (med. 104.5 vs. 49 days).

## 4. Discussion

The results of our work are a summary of the experiences of one centre in the use of ARNI in patients with HFrEF in the era before the 2021 ESC HF recommendations.

Our clinical practice regarding the inclusion of ARNI was based on the conviction of medical practitioners about the positive effect of ARNI among this group of patients and was in line with the current RCT, despite the lack of clear-cut guidelines at that time [3,4,5]. Despite the limited percentage of patients treated with ARNI in 2018–2020, we can see a significant increase in the use of ARNI over this time (from 7.8% in 2018 to almost 23% in 2020). Nordberg Backelin C. et al., in a real-world clinical setting study, showed that during the first year of the introduction of ARNI in Sahlgrenska University Hospital, only 13% of the 603 patients with HFrEF received ARNI, even though 20% of the patients were eligible and 28% were potentially eligible [6]. These values are very similar to those obtained in our study.

Our current experience with ARNI therapy was limited to extremely severe patients with HFrEF. The mean age of patients undergoing ARNI therapy was 63.6 ± 12.2 years and the mean LVEF was 23.7 ± 9.7%. Moreover, most of the patients were overweight or obese (mean BMI 28.3 ± 4.3) and had multiple comorbidities.

We can assume that patients treated with ARNI presumably had a relatively longer history of HF due to a significantly higher percentage of implanted CRT-D than the non-ARNI patients. According to our findings, patients receiving ARNI treatment suffered more frequently from CAD, the higher percentage of them was implanted and the frequency of atrial fibrillation and case history for stroke were also higher in this group. This clinical characteristic is a surrogate of the more advanced atherosclerotic disease. Our results on implanted devices and comorbidities align with the findings of the Po-Cheng Chang real-world study in Taiwan [7].

Thus, patients in clinical practice exhibit baseline characteristics of more pronounced disease severity in comparison with patients being randomized or experiencing dropout in PARADIGM-HF [1]. This could be a reason that our study results regarding clinical outcome do not directly match with PARADIGM-HF trial results where ARNI therapy reduced the rates of cardiovascular (CV) mortality or hospitalization for HF and all-cause mortality [1,2]. Another large study that compared patients with HFrEF, in a U.S. administrative claims database, treated with ARNI or ACEI/ARB from July 1, 2015 to February 2, 2018, confirms that the ARNI therapy is associated with lower risks of mortality and hospitalization compared to ACE/ARB treatment [8]. Another real-world study demonstrates that patients who received ARNI compared to patients not receiving ARNI had a lower composite risk of CV mortality and HF hospitalization [9]. Rattanavipanon W. et al. [10], in a study presenting the real-world experience of ARNI usage in Thailand on a group of 187 patients, proved that ARNI use was associated with a significant reduction of all-cause mortality and/or hospitalization for decompensated HF within 12 months. In a multicentre, noninterventional, retrospective, observational study in Turkey, where overall 779 patients with HF received ARNI, it was shown that during the use of this drug the number of hospitalizations decreased for 1 year follow-up [11]. Moreover, based on actuarial estimates of event rates and life expectancy, it is expected that ambulatory patients with HFrEF ARNI therapy will have prolonged survival by approximately 1–2 years [12]. Our results are also positive for ARNI administration; the ARNI treatment was associated with a similar number of endpoints as in the standard therapy patients, regardless of the more advanced HF and HF comorbidities. The mortality, rehospitalizations and time to the first rehospitalization in ARNI and non-ARNI groups were the same despite a significantly higher disease burden in the ARNI group. ARNI treatment was not inferior to standard treatment with ACEI/ARB.

In our study, patients treated with sacubitril/valsartan included 28 (34.1%) patients already undergoing sacubitril/valsartan therapy and 54 (65.9%) patients for whom sacubitril/valsartan was prescribed for the first time during the current hospitalization related to ADHF. This procedure is in line with the RCT results. The PIONEER-HF trial assessed the initiation of ARNI after ADHF and demonstrated that patients who received ARNI initially in the hospital had a lower incidence of HF rehospitalization or cardiovascular death than patients who started to take enalapril in the hospital and then had a delayed initiation of sacubitril/valsartan 8 weeks later [13]. Moreover, the primary results of the randomized TRANSITION study showed that the initiation of ARNI treatment in HFrEF patients following an ADHF episode, either in the hospital or shortly after discharge, is possible with approximately half of the patients reaching the target dose within 10 weeks [14].

The impact of sacubitril/valsartan therapy on the incidence of AF is still debated. According to the findings of the PARADIGM-HF trial [1] as well as Martens et al. [15], there was no impact of ARNI treatment on the incidence of AF [1,15]. Our study shows a higher frequency of AF in the ARNI group that may be a marker of the more advanced HF. Unfortunately, we have no data on the impact of ARNI implementation on AF prevalence. It should be noted that AF was associated with lower death incidence in the ARNI group.

Our study demonstrates that the independent negative predictor of death in the whole population of ARNI and non-ARNI groups was the coexistence of CAD, even though the patients had complete revascularization optimal for patients concerned. The PARADISE-MI trial, where the goal was to assess the efficacy and safety of sacubitril/valsartan compared with ramipril in a contemporary acute myocardial infarction (AMI) population, showed that ARNI treatment did not result in a significantly lower rate of CV death, HF hospitalization or outpatient HF [16]. Nevertheless, according to Morgensen et al. [17], ARNI compared to non-ARNI treatment proved to be more effective at the risk of cardiovascular death or HF hospitalization and the coronary composite of CV death, non-fatal myocardial infarction, angina hospitalization or coronary revascularization. It has also proved to reduce mortality in patients with CAD receiving ARNI treatment (20.3% ARNI vs. 17.1% non-ARNI).

Previous studies claimed that machine-learning (ML) methods can have better possibilities of detecting predictors of medication side effects [18]. Weng et al. observed that ML significantly improves the accuracy of cardiovascular risk prediction, increasing the number of patients identified who could benefit from preventive treatment, while avoiding unnecessary treatment of others [19]. In our opinion, ML methods can also open new ways of assessment of the side effects and efficacy of ARNI in the future, as in the quoted studies.

Our study emphasizes the importance of improving mechanisms to evaluate the effectiveness of HF therapies in real-world settings. The undertreatment is an international problem and has been shown repeatedly in previous studies [20]. Angiotensin receptor–neprilysin inhibitors are now a cornerstone therapy in the management of HF and resolving the effectiveness should nowadays be a top research priority.

### Study Limitations

Respective limitations should be addressed when interpreting the results. First, it is a one-centre pioneering experience; therefore, the results might reflect local practice. For instance, the higher use of CRT and ICD might be such a reflection of local practice. Only 128 patients out of 613 with HFrEF were included in the study. Additionally, the ARNI group included both newly prescribed and already prescribed ARNI patients. A low sample size may influence the nature of the cohort and limits some analysis. Secondly, the matching was limited to only two variables: age and LVEF value, which could have influenced the selection of groups that were finally analysed. We believe that, despite the simplified selection of the groups, our study provides important conclusions from the use of ARNI in real-life experience. On the other hand, the study should be considered as a preliminary observation, and the problem needs to be re-analysed based on the data of multicentre registries with an increased number of patients.

Some differences were observed in the pharmacotherapy between the ARNI and non-ARNI groups; however, the differences could be a result of the patients’ clinical profile, i.e., anticoagulation corresponded with the occurrence of atrial fibrillation.

In our database, we have information on patient deaths; however, we do not have complete data on the time of death, so we cannot evaluate prognosis using the Kaplan–Meier method and assess risk factors using Cox regression. Additionally, our 2020 experience has been influenced by the COVID-19 pandemic, which limited the number of hospitalizations due to HFrEF compared to previous years.

The strengths of our study are its innovative design, the consecutive inclusion of patients admitted with ADHF and the use of robust clinical and biological inclusion criteria based on accepted guidelines and definitions for the identification of patients with HF. Finally, our results corroborate the findings of PARADIGM-HF and correspond to a real-life representation of patients hospitalized with ADHF, characterized by comorbidities that often coexist, and the results may have external validity.

## 5. Conclusions

Our current experience in sacubitril/valsartan therapy is limited to extremely severe patients with HFrEF. The study results suggest that combined inhibition of the angiotensin receptor and neprilysin provide some additive positive value in the HFrEF treatment. Regardless of the more advanced HF and HF comorbidities, the patients treated with ARNI presented similar mortality and rehospitalizations as the patients treated by standard therapy.

## Figures and Tables

**Table 1:** Baseline characteristics of subgroups before and after matching.

Variable	Pre-Match			Post-Match		
	ARNI Subpopulation n = 82	Non-ARNI Subpopulation n = 432	Std. Mean Diff.	ARNI Subgroup n = 64	Non-ARNI Subgroup n = 64	Std. Mean Diff.
Age (years)	63.8 ± 13	67.4 ± 11.6	−0.40	63.6 ± 12.2	63.9 ± 13.4	−0.02
LVEF (%)	23.1 ± 9.2	26.8± 7.4	−0.41	23.7 ± 9.7	23.6 ± 8.3	0.01

LVEF: left ventricle ejection function; Std. Mean Diff.: standardized mean difference.

**Table 2:** The clinical characteristics of angiotensin receptor–neprilysin inhibitor (ARNI) and non-ARNI groups.

Factor	ARNI Group n = 64 n (%) or Mean ± SD or Median	non-ARNI Group n = 64 n (%) or Mean ± SD or Median	*p* Value
Age (years)	63.6 ± 12.2	63.9 ± 13.4	0.913
Sex: male	55 (85.9%)	58 (90.6%)	0.412
BMI (kg/m^2^)	28.3 ± 4.3	27.9 ± 5.3	0.680
LVEF (%)	23.7 ± 9.7	23.6 ± 8.3	0.719
NYHA class II/III/IV	II: 23 (35.9%) III: 29 (45.3%) IV: 12 (18.8%)	II: 31 (48.5%) III: 20 (31.2%) IV: 13 (20.3%)	0.237
Coronary arterial disease: ischaemic cardiomyopathy	42 (65.6%)	25 (39.1%)	0.003
CIED (ICD + CRT-D)	40 (62.5%)	26 (40.6%)	0.014
ICD	20 (31.2%)	16 (25.0%)	0.434
CRT-D	20 (31.2%)	10 (15.6%)	0.038
Atrial fibrillation	29 (45.3%)	16 (25.0%)	0.002
Mitral regurgitation (II/III)	23 (35.9%)	26 (40.6%)	0.587
Tricuspid regurgitation (II/III)	15 (23.4%)	20 (31.3%)	0.323
Concomitant diseases:			
• Chronic kidney disease	21 (32.8%)	18 (28.1%)	0.606
• Hypertension	33 (51.6%)	36 (56.3%)	0.596
• Diabetes	18 (28.1%)	17 (26.6%)	0.843
• Lipid disorders	22 (34.4%)	26 (40.6%)	0.467
• POAD	9 (14.1%)	4 (6.3%)	0.145
• History of stroke	11 (17.2%)	3 (4.7%)	0.024
• Respiratory diseases (asthma, COPD)	7 (10.9%)	12 (18.8%)	0.216
Medications			
• MRA	54 (84.4%)	51 (79.7%)	0.061
• Β-blockers	54 (84.4%)	57 (89.1%)	0.069
• Calcium antagonist (dihydropyridine derivatives)	5 (7.8%)	4 (6.3%)	0.030
• Loop diuretic	61 (95.3%)	56 (87.5%)	0.138
• Thiazide	15 (23.4%)	19 (29.7%)	0.425
• Statin	41 (64.1%)	38 (59.4%)	0.295
• Fibrate	2 (3.1%)	2 (3.1%)	----
• Ezetimibe	4 (6.3%)	0	0.043
• VKA	11 (17.2%)	12 (18.8%)	0.053
• NOAC	29 (45.3%)	15 (23.4%)	0.017
• Acetylsalicylic acid	22 (34.4%)	34 (53.1%)	0.033
• P2Y12 inhibitors	14 (21.9%)	14 (21.9%)	---
• Insulin	5 (7.8%)	4 (6,3%)	0.731
• Metformin	7 (10.9%)	9 (14.1%)	0.594
• Sulfonylureas	4 (6.3%)	2 (3.1%)	0.405
• DDP-4 inhibitors	0	1 (1.6%)	0.317
• SGLT-2 inhibitors	6 (9.4%)	3 (4.7%)	0.302
Clinical endpoints			
Rehospitalization	12 (18.8%) 10 patients: 1 time 2 patients: 3 times	13 (20.3%) 12 patients: 1 time 1 patient: 4 times	0.722
Time to the first rehospitalization (days)	199.3 ± 222.1 Med.: 114.5 (71; 268.5)	165.3 ± 138.2 Med.: 126 (83.8; 209.8)	0.978
Number of rehospitalizations	16 (25.0%)	16 (25.0%)	---
Deaths	18 (28.1%)	18 (28.1%)	---

ARNI: angiotensin receptor–neprilysin inhibitor; BMI: body mass index; CIED: cardiovascular implantable electronic device; COPD: chronic obstructive pulmonary disease; CRT-D: defibrillator with cardiac resynchronization therapy; ICD: implantable cardioverter defibrillator; LVEF: left ventricle ejection function; MRA: mineralocorticoid receptor antagonist; NOAC: non-VKA oral anticoagulants; NYHA: New York Heart Association; POAD: peripheral occlusive artery disease; VKA: vitamin K antagonists.

**Table 3:** The clinical characteristics of the dead and alive patients from ARNI group.

Factor	Deaths n = 18 n (%) or Mean ± SD or Median	Alive n = 46 n (%) or Mean ± SD or Median	*p* Value
Age (years)	66.6 ± 13.9	62.5 ± 11.4	0.007
Sex: male	17 (94.4%)	38 (82.6%)	0.150
BMI (kg/m^2^)	29.6 ± 5.4	27.8 ± 3.8	0.140
LVEF (%)	20.2 ± 7.1	25.1 ± 10.3	0.048
NYHA class II/III/IV	II: 4 (22.2%) III: 8 (44.4%) IV: 6 (33.3%)	II: 19 (41.3%) III: 21 (45.7%) IV: 6 (13.0%)	0.049
Coronary arterial disease: ischaemic cardiomyopathy	15 (83.3%)	27 (58.7%)	0.064
CIED (ICD + CRT-D)	12 (66.7%)	28 (60.9%)	0.669
ICD	4 (22.2%)	16 (34.8%)	0.333
CRT-D	8 (44.4%)	12 (26.1%)	0.158
Atrial fibrillation	4 (22.2%)	25 (54.3%)	0.021
Mitral regurgitation (II/III)	6 (33.3%)	17 (37%)	0.788
Tricuspid regurgitation (II/III)	5 (27.8%)	10 (21.7%)	0.611
Concomitant diseases:			
• Chronic kidney disease	8 (44.4%)	13 (28.3%)	0.219
• Hypertension	10 (55.6%)	23 (50%)	0.692
• Diabetes	7 (38.9%)	11 (23.9%)	0.235
• Lipid disorders	4 (22.2%)	18 (39.1%)	0.204
• POAD	4 (22.2%)	5 (10.9%)	0.244
• History of stroke	2 (11.1%)	9 (19.6%)	0.424
• Respiratory diseases (asthma, COPD)	4 (22.2%)	3 (6.5%)	0.073
Clinical endpoints			
• Rehospitalization	6 (33.3%) 5 patients: 1 time 1 patient: 3 times	6 (13.0%) 5 patients: 1 time 1 patient: 3 times	0.137
• Time to the first rehospitalization (days)	221.5 ± 295.9 Med.:104.5 (65; 147)	187.2 ± 141.15 Med.:163.5 (77; 311)	0.749
• Number of rehospitalizations	8 (44.4%)	8 (17.4%)	0.097

ARNI: angiotensin receptor–neprilysin inhibitor; BMI: body mass index; LVEF: left ventricle ejection function; NYHA: New York Heart Association; CIED: cardiovascular implantable electronic device; ICD: implantable cardioverter defibrillator; CRT-D: defibrillator with cardiac resynchronization therapy; POAD: peripheral occlusive artery disease; COPD: chronic obstructive pulmonary disease.

**Table 4:** The clinical characteristics of the dead and alive patients from non-ARNI group.

Factor	Deaths n = 18 n (%) or Mean ± SD or Median	Alive n = 46 n (%) or Mean ± SD or Median	*p* Value
Age (years)	61.9 ± 17.5	64.7 ± 11.6	0.457
Sex: male	18 (100%)	40 (87%)	0.110
BMI (kg/m^2^)	28 ± 4.7	27.9 ± 5.6	0.960
LVEF (%)	20.4 ± 7.5	24.8 ± 8.4	0.050
NYHA class II/III/IV	II: 4 (22.2%) III: 8 (44.4%) IV: 6 (33.3%)	II:27 (58.7%) III: 12 (26.1%) IV: 7 (15.2%)	0.030
Coronary arterial disease: ischaemic cardiomyopathy	6 (33.3%)	19 (41.3%)	0.560
CIED (ICD + CRT-D)	8 (44.4%)	18 (39.1%)	0.699
ICD	3 (16.7%)	13 (28.3%)	0.340
CRT-D	5 (27.8%)	5 (10.9%)	0.097
Atrial fibrillation	4 (22.2%)	12 (26.1%)	0.750
Mitral regurgitation (II/III)	10 (55.6%)	16 (34.8%)	0.131
Tricuspid regurgitation (II/III)	9 (50%)	11 (23.9%)	0.045
Concomitant diseases:			
• Chronic kidney disease	8 (44.4%)	10 (21.7%)	0.080
• Hypertension	5 (27.8%)	31 (67.4%)	0.004
• Diabetes	4 (22.2%)	13 (28.3%)	0.625
• Lipid disorders	6 (33.3%)	20 (43.5%)	0.461
• POAD	2 (11.1%)	2 (4.3%)	0.318
• History of stroke	1 (5.6%)	2 (4.3%)	0.838
• Respiratory diseases (asthma, COPD)	4 (22.2%)	8 (17.4%)	0.659
Clinical endpoints			
• Rehospitalization	4 (22.2%) 3 patients: 1 time 1 patient: 4 time	9 (19.6%) 9 patients: 1 time	0.848
• Time to the first rehospitalization (days)	158.5 ± 239.1 Med.: 49 (26.5; 290.5)	168.3 ± 84.7 Med.: 130 (100.8; 209.8)	0.165
• Number of rehospitalizations	7 (38.9%)	9 (19.6%)	0.231

ARNI: angiotensin receptor–neprilysin inhibitor; BMI: body mass index; LVEF: left ventricle ejection function; NYHA: New York Heart Association; CIED: cardiovascular implantable electronic device; ICD: implantable cardioverter defibrillator; CRT-D: defibrillator with cardiac resynchronization therapy; POAD: peripheral occlusive artery disease; COPD: chronic obstructive pulmonary disease.

## Data Availability

The data presented in this study are available on request from the corresponding author. The data are not publicly available due to confidentiality of the research.
